# Parasitic Contamination of Commonly Consumed Fresh Leafy Vegetables in Benha, Egypt

**DOI:** 10.1155/2014/613960

**Published:** 2014-06-16

**Authors:** Maysa Ahmad Eraky, Samia Mostafa Rashed, Mona El-Sayed Nasr, Azza Mohammed Salah El-Hamshary, Amera Salah El-Ghannam

**Affiliations:** Parasitology Department, Faculty of Medicine, Benha University, Benha 13518, Egypt

## Abstract

This study evaluated the degree of parasitic contamination of vegetables which are commercialized and consumed fresh in Benha, Egypt. It included 530 vegetables: lettuce, watercress, parsley, green onion, and leek. Vegetables were collected randomly from markets within Benha. Samples were washed in saline, and the resulting washing solution was filtered and centrifuged to concentrate the parasitic stages. Sediments and supernatants were examined by iodine and modified Ziehl-Neelsen stained smears. Intestinal parasites were detected in 157/530 (29.6%) samples. *Giardia lamblia* cysts were the most prevalent parasite (8.8%) followed by *Entamoeba* spp. cysts (6.8%), *Enterobius vermicularis* eggs (4.9%), various helminth larvae (3.6%), *Hymenolepis nana* eggs (2.8%), *Hymenolepis diminuta* eggs (2.1%), and *Ascaris lumbricoides* eggs (0.6%). The highest contaminated vegetable was lettuce (45.5%) followed by watercress (41.3%), parsley (34.3%), green onion (16.5%), and leek (10.7%). These results indicate a significant seasonal variation (*P* < 0.05), with highest prevalence in summer (49%) and the lowest in winter (10.8%). These findings provide evidence for the high risk of acquiring parasitic infection from the consumption of raw vegetables in Benha, Egypt. Effective measures are necessary to reduce parasitic contamination of vegetables.

## 1. Introduction

Intestinal parasites are among the main public health problems around the world especially in tropical and subtropical countries [1]. In recent years, there has been an increase in the number of reported cases of foodborne illness linked to consumption of fresh vegetables. The consumption of raw vegetables plays a major epidemiological role in the transmission of parasitic foodborne diseases [2].

Intestinal parasites such as* Cryptosporidium* spp.,* Giardia lamblia*,* Entamoeba histolytica*,* Ascaris lumbricoides*, hookworms,* Enterobius vermicularis*,* Trichuris trichiura*,* Toxocara *spp.,* Hymenolepis *spp.,* Taenia *spp.,* Fasciola *spp., and members of the family Trichostrongylidae could infect humans as a result of consumption of contaminated, uncooked, or improperly washed vegetables [3].

Outbreaks of human infections associated with consumption of raw fruits and vegetables have occurred with increased frequency during the past decade. Factors contributing to this increase may include changes in agronomic and processing practices, an increase in per capita consumption of raw or minimally processed fruits and vegetables, increased international trade and distribution, and an increase in the number of immunocompromised consumers. A general lack of efficacy of sanitizers in removing or killing pathogens on raw fruits and vegetables has been attributed, in part, to their inaccessibility to locations within structures and tissues that may harbour pathogens [4].

Epidemiological studies have also indicated that, in areas of the world where parasitic diseases are endemic in the population and where wastewater is used to irrigate vegetables which are eaten raw, the consumption of wastewater irrigated vegetables without proper washing may lead to parasitic infection [5].

Different parasitic stages can contaminate vegetables. The most likely hypothesis of contamination is that it occurred before harvest while still on the plants in fields, either by contaminated manure, sewage, irrigation water, and wastewater from livestock operations or directly from wild and domestic animals [6] or during harvesting, transport, processing, distribution, and marketing or even at home [7].

In developing countries, because of inadequate or even nonexisting systems for routine diagnosis and monitoring or reporting for many of the foodborne pathogens, most outbreaks caused by contaminated vegetables go undetected and the incidence of their occurrence in food is underestimated [8].

Many studies have been done to evaluate the role of raw vegetables in transmission of intestinal parasites, such as in Alexandria, Egypt [7, 9], Tripoli, Libya [10], Riyadh, Saudi Arabia [2], Iraq [11], Tehran [12], and Philippines [13]. The results in these studies indicated different levels of parasitic contamination of raw vegetables.

To our knowledge, there is no previously published data about the contamination of fresh leafy vegetables in Benha. Therefore, this study provides important information to stake holders on the potential contamination of vegetables.

## 2. Materials and Methods

The present study was carried out during the period from September, 2012, to August, 2013. The study included 530 vegetable samples, comprised of five types of leafy vegetables: lettuce (101), watercress (116), parsley (102), green onion (103), and leek (108).

### 2.1. Procedure for Sample Collection, Preparation, and Washing

Fresh samples were collected randomly from Benha markets. Each vegetable sample was placed in a separate nylon bag and labelled with a unique number and date of collection. Approximately 200 gm of each vegetable was soaked (for fifteen minutes) in one liter of physiological saline, followed by vigorous shaking with the aid of a mechanical shaker for 15 minutes. Vegetable sample was removed and the remaining wash solution was left for 10 hours to sediment. The top layer was discarded and the remaining wash solution was filtered through a sieve (425 um pore size) to remove large debris and then centrifuged at 2000 rpm (447 ×g) for 15 min. The supernatant was decanted into another tube to be examined by floatation, and a few drops of the sediment were placed on glass slides and examined for parasites [10].

### 2.2. Examination of Samples

The supernatant was examined by a zinc sulphate flotation technique to detect various helminth eggs and larvae and protozoal cysts [14]. The sediment was mixed and stained and unstained smears were examined for parasites. For the unstained smear, a drop of the sediment was applied onto a freshly clean slide, and a cover slip was gently placed to avoid air bubbles and flooding. The preparation was then examined under a light microscope using multiple objectives: (×10), (×40), and (×100) [15].

An iodine stained smear was prepared by adding a small drop of Lugol's iodine solution prior to the placing of a cover slip to a slide similarly prepared for the unstained smear.

Modified Ziehl-Neelsen stained smears were prepared for detection of coccidian protozoal oocysts including* Cryptosporidium *spp.,* Isospora belli, *and* Cyclospora cayetanensis* [16].

### 2.3. Statistical Analysis

Data and laboratory findings were organized and analyzed using the computer program statistical package for social science (SPSS) version 16. In the statistical comparison between the groups, the significance of difference was analysed using the *Z* test to compare the proportion between two groups of qualitative data. Intergroup comparison of categorical data was performed by using chi square test (*χ* ^2^ value) or fisher exact test (FET). A *P*value < 0.05 was considered statistically significant.

## 3. Results and Discussion

High incidences of intestinal parasites have been found in communities that consume raw vegetables, especially that they are cultivated on farms fertilized with untreated human and animal fertilizers [17]. Furthermore, preference for eating raw or slightly cooked vegetables to protect heat-labile nutrients may increase the risk of foodborne infections [18]. Because Egypt is among the areas that have significant parasitic infections, identifying the source of infection, methods of transmission, spread of such infections, and methods of prevention warrant priority. A limited number of studies have been conducted in this country to evaluate the degree of parasitic contamination on vegetables. Our study is the first that was carried out to determine the extent of parasitic contamination of some of the commonly consumed raw leafy vegetables distributed in markets of Benha City, Qalyubia Governorate, Egypt.

This study showed a considerably high level of contamination of green vegetables involving intestinal parasites (29.6%), with lettuce being the highest contaminated vegetable (45.5%), followed by watercress (41.3%), parsley (34.3%), green onion (16.5%), and leek as the least contaminated (10.7%) (Table 1). This finding was consistent with previous reports from Alexandria, Egypt [7], and in Ardabil, Iran [19], where the contamination rates were (31.7%) and (29%), respectively. Higher contamination rates were detected in Ghana (36%) [20], Nigeria (36%) [5], Tripoli, Libya (58%) [10], and Kenya (75.9%) [21], and the highest rate was detected in Khorramabad, Iran (79%) [22]. Lower rates of contamination in the Middle East were detected in Riyadh, Saudi Arabia (16.2%) [2], and Turkey (6.3%) [23]. This variation in contamination rates may, in part, be due to the differences in shape and surface of vegetables. Green leafy vegetables such as lettuce, watercress, and parsley have uneven surfaces that probably facilitate sticking of parasitic eggs, cysts, and oocysts more readily, either at the farm or when washed with contaminated water. However, vegetables with smooth surface such as leek and green onion had the lowest prevalence rates [5, 7].

**Figure 1:**
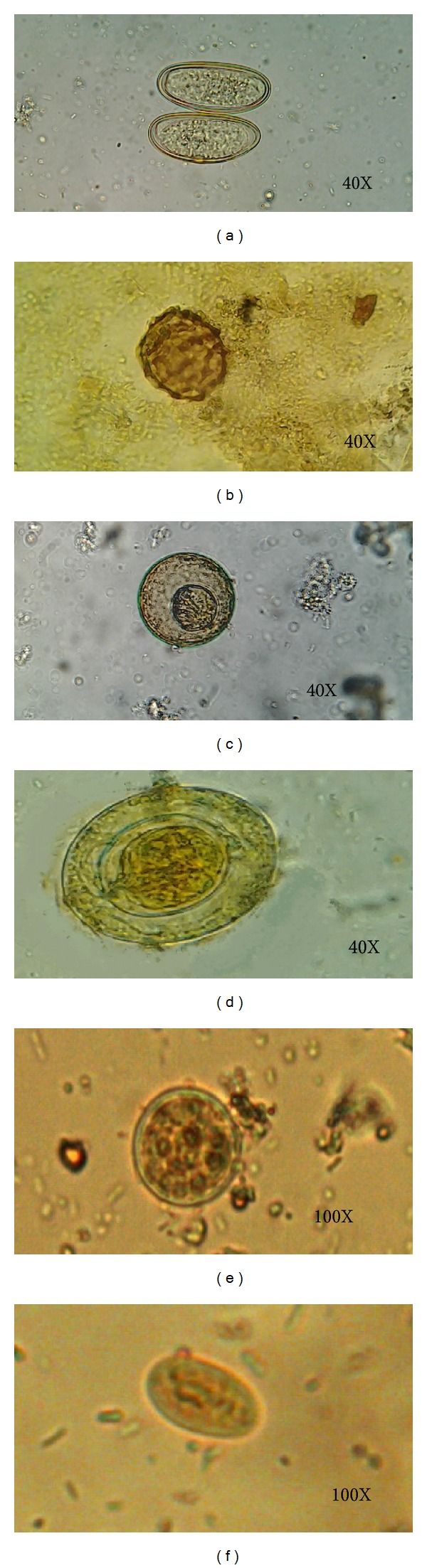
Some of the detected parasitic stages contaminating raw leafy vegetables: (a)* Enterobius vermicularis *eggs (40X), (b)* Ascaris lumbricoides *eggs (40X), (c)* Hymenolepis diminuta *eggs (40X), (d)* Hymenolepis nana *eggs (40X), (e)* Entamoeba coli *cyst (100X), and (f)* Giardia lamblia *cyst (100X).

*Giardia lamblia *cysts were the most prevalent parasitic stage contaminating green vegetables (8.8%) (Table 2; Figure 1) with lettuce being most contaminated (15.8%). The finding of this study was similar to previous reports in Alexandria, Egypt [7, 9], and Shahrekord, Iran [24].

The second most prevalent parasitic stage was* Entamoeba *spp. cyst (6.8%), with lettuce being most contaminated (13.9%) (Table 2; Figure 1). Similar results were detected in Shahrekord, Iran (9.2%) [18], Khorramabad, Iran (11.3%) [22], and Alexandria, Egypt (11.2%) [9]. However, higher rates of* Entamoeba coli *contamination were detected (19.04%) in South Western Saudi Arabia, and* Entamoeba histolytica *was 3.1% in the same study [25]. Higher rates were detected in Riyadh, Saudi Arabia, and Gaza Governorate, (35.5% and 37.5%, resp.) [2, 26]. Lower rates were detected in Nigeria (14%) [5] and Tabriz, Iran (8%) [27]. In Manila, Philippines,* Entamoeba histolytica *was 0.6% and* Entamoeba coli *was 2.5% [13].

*Enterobius vermicularis *eggs were detected in 4.9% (Table 2; Figure 1) with watercress samples being most contaminated (9.5%). It was similar to studies conducted in other countries: in Manila, Philippines, as* Enterobius vermicularis *eggs were 4.5% [13], In Khorramabad, Iran, the detected rate was 5.1% [22]. Higher rates were detected in South Western Saudi Arabia (6.3%) and Zahedan, Iran (8.1%), respectively [25, 28]. Lower contamination rate (0.8%) was detected in Nigeria [29] and (0.9%) in Turkey [23].

In this study, unidentified helminth larvae were detected in 3.6% of vegetable samples (Table 2), with lettuce samples being most contaminated (5.9%). Garedaghi et al. [27] reported higher rate of free living larvae (6%) in imported vegetable and (7%) in native vegetable. Free living larvae were detected in 40% in Khorramabad, Iran [22].

*Hymenolepis nana *eggs were detected in 2.8% with the highest rate in watercress samples (6%) (Table 2, Figure 1), while* Hymenolepis diminuta *eggs were detected in 2.1% with lettuce samples being most contaminated (3.9%). Similar rates were detected in Alexandria, Egypt (2.6%), and lettuce was the main type of vegetable contaminated with these parasitic eggs (6.7%) [7]. Similar rate (2.4%) was also detected in Libya. In Zahedan, Iran, the contamination rate was 5%, while* Hymenolepis diminuta *was detected in 2% of examined samples in the same study [28]. This rate was increased to 14.5% in Riyadh, Saudi Arabia [2]. In Qazvin, Iran,* Hymenolepis nana *eggs were detected in 0.5% only of tested samples and it was the least parasite contaminating the green vegetables.

*Ascaris lumbricoides *eggs were the least detected parasitic stages contaminating green vegetables (0.6%) in this study (Table 2; Figure 1). This parasite was detected in lettuce (1.9%) and watercress (0.8%), but not in parsley, leek, or green onion samples (0.0%). The rates of contamination with* Ascaris *eggs in vegetables in Iran were 2% in Ardabil [19], 2.5% in Jiruft [30], 2.3% in Qazvin [31], and 6.1% in Zahedan [28] and 5.8% in a study conducted in Khorramabad, where the highest contamination rate with this parasite was detected in green onion samples (12.7%) [22]. In Plateau State, Nigeria, the contamination rate was 2.4% [32].

The presence of helminth eggs in different vegetables may be related to either contamination of soil or contamination of irrigating water [33]. Although contamination of vegetables may occur in a variety of ways, it is mainly associated with the water used for irrigation. The use of sewage contaminated water for irrigation of vegetables is a common practice in developing countries including Egypt [7].

The broad range in prevalence could be attributed to many factors. These may include geographical location, type and number of samples examined, methods used for detection of the intestinal parasites, type of water used for irrigation, and postharvesting handling methods of such vegetables which are different from one country to another. Other factors that can affect parasitic transmission may also include population related hygienic habits, sanitary facilitations, climatic conditions, and range of foodborne parasites endemicity in certain countries.

Considering seasonal variability (Table 3), this study indicates that the rate of parasitic contamination in vegetable samples was the highest in summer (49%) and the lowest in winter (10.8%). The number of contaminated samples in summer and autumn was significantly high (*P* > 0.05). The number of contaminated samples in spring was also statistically significant (*P* > 0.05) when compared to the number of contaminated samples in winter which was statistically insignificant (*P* > 0.05). Our findings were consistent with previous studies that reported higher rates of parasitic contamination of raw vegetables during warm seasons than those during cold seasons [2, 7, 19].

Another study in Hanoi, Vietnam, showed that the number of eggs recovered from vegetables was higher in the dry season (78%) than in the rainy season (22%) of total number of eggs recovered [34]. The number of eggs recovered was higher in the dry than in the rainy seasons and it is assumed that eggs on the surface of vegetables are washed away by rain [34]. It has been determined that the excretion of parasite's eggs to environment by human or animals is high in warm seasons compared to cold seasons [7, 35].

## 4. Conclusion

Our results clearly show that raw leafy vegetables consumed by people are quite often contaminated with parasites. These types of vegetables should be considered as a potential source of parasitic contamination in Benha. These findings underscore the public health implication of consumers of these vegetables being at high risk of infection with giardiasis, amoebiasis, enterobiasis, hymenolepiasis, ascariasis, and likely others. These parasites may be acquired through the consumption of these vegetables, especially when not hygienically grown and adequately prepared before consumption. Inhabitants of this region should be informed how to properly disinfect these vegetables before consuming them raw.

## Figures and Tables

**Table 1:** Prevalence rate of parasitic contamination on fresh leafy vegetables.

	Vegetable											
	Lettuce (101)		Watercress (116)		Parsley (102)		Green onion (103)		Leek (108)		Total examined (530)	
	Number	%	Number	%	Number	%	Number	%	Number	%	Number	%
Positive	46	45.5	48	41.3	35	34.3	17	16.5	11	10.7	157	29.6
Negative	55	54.5	68	58.7	67	65.6	86	83.5	97	89.8	373	70.3
*Z* test	0.899		1.89		3.34		9.16		13.68		10.27	
*P* value	0.18		0.03∗		0.001∗		0.001∗		0.001∗ S		0.001∗	

*Significant *P* value (*P* < 0.05).

**Table 2:** Distribution of parasitic contamination on fresh leafy vegetables.

Parasites	Number	% of total examined samples (530)	% of total positive samples (157)	Lettuce (101)		Watercress (116)		Parsley (102)		Green onion (103)		Leek (108)		Total	
				Number of +ve	%	Number of +ve	%	Number of +ve	%	Number of +ve	%	Number of +ve	%	Number of +ve	%
*Enterobius vermicularis* eggs	26	4.9	16.5	3	11.5	11	42.3	7	26.9	4	15.4	1	3.9	26	100
*Hymenolepis nana* eggs	15	2.8	9.5	1	6.7	7	46.7	4	26.7	3	20.0	0	0.0	15	100
*Hymenolepis diminuta* eggs	11	2.1	7	4	36.3	3	27.3	2	18.2	1	9.1	1	9.1	11	100
*Ascaris lumbricoid*es eggs	3	0.6	1.9	2	66.7	1	33.3	0	0.0	0	0.0	0	0.0	3	100
Helminth larvae	19	3.6	12.1	6	31.5	4	21.1	2	10.5	3	15.8	4	21.1	19	100
*Entamoeba* spp. cyst	36	6.8	22.9	14	38.9	9	25.0	8	22.2	2	5.6	3	8.3	36	100
*Giardia lamblia* cyst	47	8.8	29.9	16	34.0	13	27.7	12	25.5	4	8.5	2	4.3	47	100
Total	**157**	**29.6**	**100**	**46**	**45.5**	**48**	**41.3**	**35**	**34.3**	**17**	**16.5**	**11**	**10.2**		
FET	25.9														
*P* value	0.36 NS														

**Table 3:** Number and percentage of contaminated samples with intestinal parasites in different seasons in the examined fresh leafy vegetables.

Vegetable		Spring (130)		Summer (155)		Autumn (115)		Winter (130)		*χ* ^2^ test	*P* value
		Number	%	Number	%	Number	%	Number	%		
Lettuce	+ve	9	36.0	19	70.4	12	57.1	6	21.4	15.33	0.001∗
Watercress	+ve	13	40.6	23	69.7	8	30.8	4	16.0	18.76	0.001∗
Parsley	+ve	8	34.8	17	51.5	7	33.3	3	12.0	9.87	0.019∗
Green onion	+ve	3	11.1	9	30.0	4	19.1	1	4.0	7.47	0.058
Leek	+ve	2	8.7	8	25.0	1	3.9	0	0.0	11.94	0.007∗
Total	**+ve**	**35**	**26.9**	**76**	**49.0**	**32**	**27.8**	**14**	**10.8**	**50.81**	**0.001** ∗
Test		12.14		22.38		17.7		8.5			
*P* value		0.016∗		0.001∗		0.001∗		0.075			

+ve: number of contaminated samples with intestinal parasites/season, −ve: number of noncontaminated samples with intestinal parasites/season, and %: percentage of contaminated samples with intestinal parasites for each type of green vegetable/season.

∗significant difference between examined vegetable groups in different seasons, Rows or horizontal: the same vegetable type difference in different seasons, Columns or vertical: difference between different vegetables in the same season.
